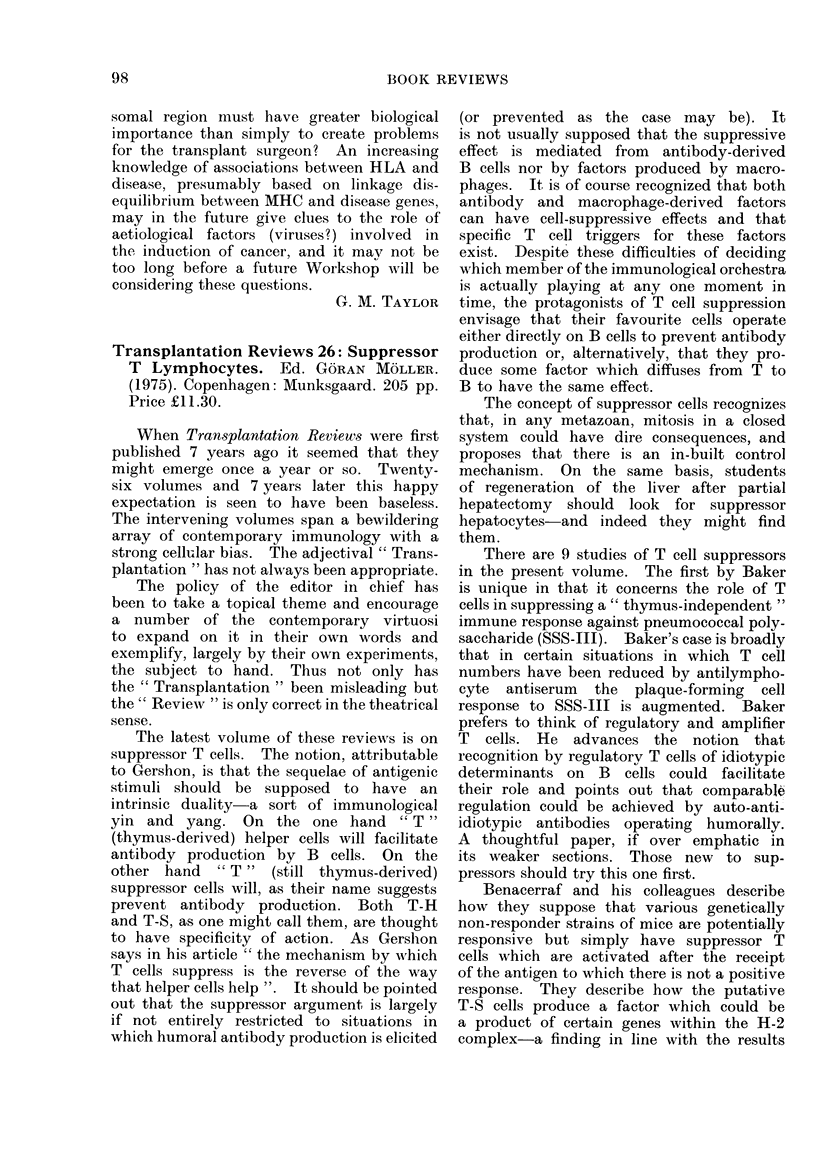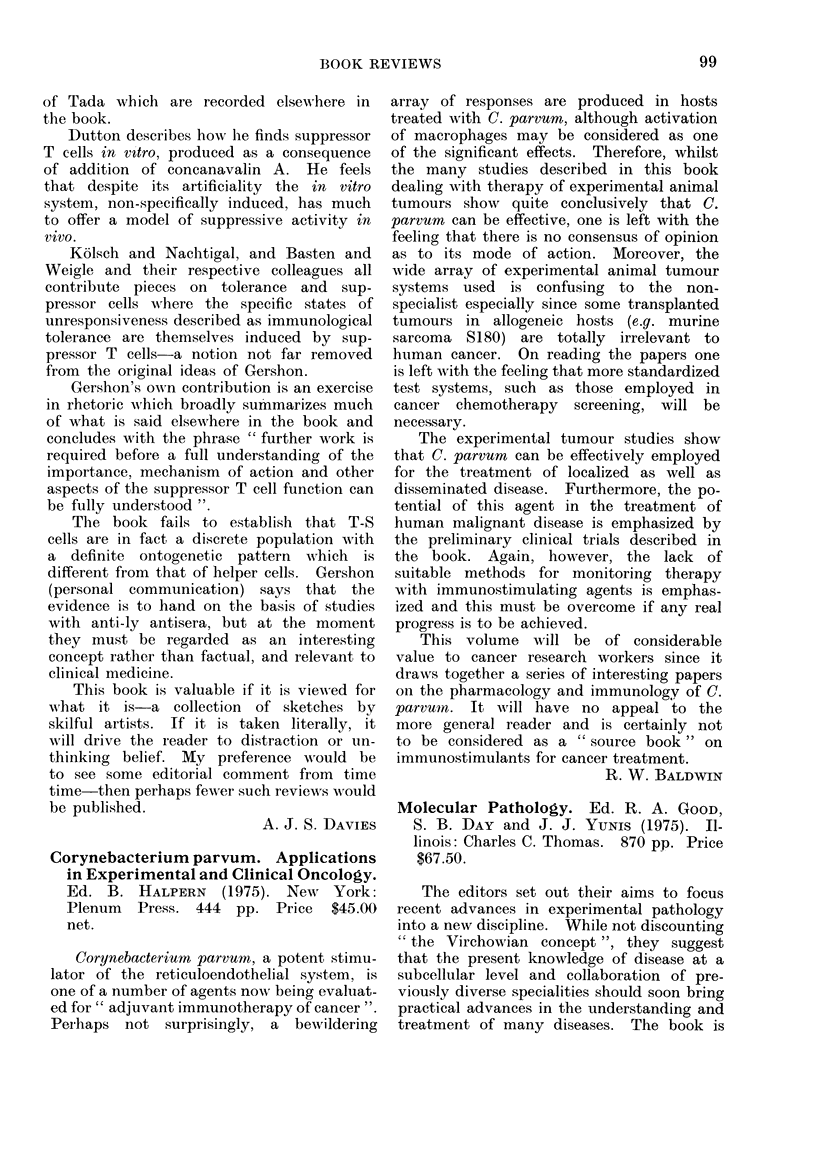# Transplantation Reviews 26: Suppressor T Lymphocytes

**Published:** 1976-07

**Authors:** A. J. S. Davies


**Transplantation Reviews 26: Suppressor T Lymphocytes.** Ed. GÖRAN MÖLLER. (1975). Copenhagen: Munksgaard. 205 pp. Price £11.30.

When *Transplantation Reviews* were first published 7 years ago it seemed that they might emerge once a year or so. Twenty-six volumes and 7 years later this happy expectation is seen to have been baseless. The intervening volumes span a bewildering array of contemporary immunology with a strong cellular bias. The adjectival " Transplantation " has not always been appropriate.

The policy of the editor in chief has been to take a topical theme and encourage a number of the contemporary virtuosi to expand on it in their own words and exemplify, largely by their own experiments, the subject to hand. Thus not only has the " Transplantation " been misleading but the " Review " is only correct in the theatrical sense.

The latest volume of these reviews is on suppressor T cells. The notion, attributable to Gershon, is that the sequelae of antigenic stimuli should be supposed to have an intrinsic duality—a sort of immunological yin and yang. On the one hand " T " (thymus-derived) helper cells will facilitate antibody production by B cells. On the other hand " T " (still thymus-derived) suppressor cells will, as their name suggests prevent antibody production. Both T-H and T-S, as one might call them, are thought to have specificity of action. As Gershon says in his article " the mechanism by which T cells suppress is the reverse of the way that helper cells help ". It should be pointed out that the suppressor argument is largely if not entirely restricted to situations in which humoral antibody production is elicited (or prevented as the case may be). It is not usually supposed that the suppressive effect is mediated from antibody-derived B cells nor by factors produced by macrophages. It is of course recognized that both antibody and macrophage-derived factors can have cell-suppressive effects and that specific T cell triggers for these factors exist. Despite these difficulties of deciding which member of the immunological orchestra is actually playing at any one moment in time, the protagonists of T cell suppression envisage that their favourite cells operate either directly on B cells to prevent antibody production or, alternatively, that they produce some factor which diffuses from T to B to have the same effect.

The concept of suppressor cells recognizes that, in any metazoan, mitosis in a closed system could have dire consequences, and proposes that there is an in-built control mechanism. On the same basis, students of regeneration of the liver after partial hepatectomy should look for suppressor hepatocytes—and indeed they might find them.

There are 9 studies of T cell suppressors in the present volume. The first by Baker is unique in that it concerns the role of T cells in suppressing a " thymus-independent " immune response against pneumococcal polysaccharide (SSS-III). Baker's case is broadly that in certain situations in which T cell numbers have been reduced by antilymphocyte antiserum the plaque-forming cell response to SSS-III is augmented. Baker prefers to think of regulatory and amplifier T cells. He advances the notion that recognition by regulatory T cells of idiotypic determinants on B cells could facilitate their role and points out that comparable regulation could be achieved by auto-anti-idiotypic antibodies operating humorally. A thoughtful paper, if over emphatic in its weaker sections. Those new to suppressors should try this one first.

Benacerraf and his colleagues describe how they suppose that various genetically non-responder strains of mice are potentially responsive but simply have suppressor T cells which are activated after the receipt of the antigen to which there is not a positive response. They describe how the putative T-S cells produce a factor which could be a product of certain genes within the H-2 complex—a finding in line with the results of Tada which are recorded elsewhere in the book.

Dutton describes how he finds suppressor T cells *in vitro*, produced as a consequence of addition of concanavalin A. He feels that despite its artificiality the *in vitro* system, non-specifically induced, has much to offer a model of suppressive activity *in vivo*.

Kölsch and Nachtigal, and Basten and Weigle and their respective colleagues all contribute pieces on tolerance and suppressor cells where the specific states of unresponsiveness described as immunological tolerance are themselves induced by suppressor T cells—a notion not far removed from the original ideas of Gershon.

Gershon's own contribution is an exercise in rhetoric which broadly summarizes much of what is said elsewhere in the book and concludes with the phrase " further work is required before a full understanding of the importance, mechanism of action and other aspects of the suppressor T cell function can be fully understood ".

The book fails to establish that T-S cells are in fact a discrete population with a definite ontogenetic pattern which is different from that of helper cells. Gershon (personal communication) says that the evidence is to hand on the basis of studies with anti-ly antisera, but at the moment they must be regarded as an interesting concept rather than factual, and relevant to clinical medicine.

This book is valuable if it is viewed for what it is—a collection of sketches by skilful artists. If it is taken literally, it will drive the reader to distraction or unthinking belief. My preference would be to see some editorial comment from time time—then perhaps fewer such reviews would be published.

A. J. S. Davies